# Young People Seeking Help Online for Mental Health: Cross-Sectional Survey Study

**DOI:** 10.2196/13524

**Published:** 2019-08-26

**Authors:** Claudette Pretorius, Derek Chambers, Benjamin Cowan, David Coyle

**Affiliations:** 1 School of Computer Science University College Dublin Dublin Ireland; 2 Health Service Executive Cork Ireland; 3 School of Information and Communication Studies University College Dublin Dublin Ireland

**Keywords:** mental health, eHealth, mHealth, Internet, help-seeking behavior, health literacy, young adults, survey and questionnaires

## Abstract

**Background:**

Young people are particularly vulnerable to experiencing mental health difficulties, but very few seek treatment or help during this time. Online help-seeking may offer an additional domain where young people can seek aid for mental health difficulties, yet our current understanding of how young people seek help online is limited.

**Objective:**

This was an exploratory study which aimed to investigate the online help-seeking behaviors and preferences of young people.

**Methods:**

This study made use of an anonymous online survey. Young people aged 18-25, living in Ireland, were recruited through social media ads on Twitter and Facebook and participated in the survey.

**Results:**

A total of 1308 respondents completed the survey. Many of the respondents (80.66%; 1055/1308) indicated that they would use their mobile phone to look online for help for a personal or emotional concern. When looking for help online, 82.57% (1080/1308) of participants made use of an Internet search, while 57.03% (746/1308) made use of a health website. When asked about their satisfaction with these resources, 36.94% (399/1080) indicated that they were satisfied or very satisfied with an Internet search while 49.33% (368/746) indicated that they were satisfied or very satisfied with a health website. When asked about credibility, health websites were found to be the most trustworthy, with 39.45% (516/1308) indicating that they found them to be trustworthy or very trustworthy. Most of the respondents (82.95%; 1085/1308) indicated that a health service logo was an important indicator of credibility, as was an endorsement by schools and colleges (54.97%; 719/1308). Important facilitators of online help-seeking included the anonymity and confidentiality offered by the Internet, with 80% (1046/1308) of the sample indicating that it influenced their decision a lot or quite a lot. A noted barrier was being uncertain whether information on an online resource was reliable, with 55.96% (732/1308) of the respondents indicating that this influenced their decision a lot or quite a lot.

**Conclusions:**

Findings from this survey suggest that young people are engaging with web-based mental health resources to assist them with their mental health concerns. However, levels of satisfaction with the available resources vary. Young people are engaging in strategies to assign credibility to web-based resources, however, uncertainty around their reliability is a significant barrier to online help-seeking.

## Introduction

Globally, there is a growing recognition of the public health challenge associated with mental disorders [1-4]. Particularly, the mental health of young people is becoming of increasing concern [5,6]. It has been recognized that young people are especially vulnerable to experiencing mental health difficulties, with very few seeking treatment or help during this time [5,6]. A systematic review by Ibrahim et al [7] found that students, most of whom were between the ages of 18 to 25, experienced higher rates of depression than other age groups from the general population. This age group faces unique challenges and stressors as they transition into adulthood [8], as young people are expected to learn adult responsibilities, and many experience high levels of distress as they potentially make sense of numerous changes taking place in their lives [9]. The personal and emotional concerns associated with this stage of a young person’s life, and how they seek help for these worries, are of critical concern.

Help-seeking for mental health difficulties is often understood to be an adaptive coping method where an individual engages in behavior that communicates their distress to others with the goal of getting help in the form of understanding, advice, information, treatment or general support [10,11]. Help-seeking is a complicated process influenced by a person’s attitudes, preferences and goals. For this reason, many people make use of a multitude of sources of help [12-14]. Previous research has found that engaging in help-seeking behavior, both formal and informal, is an important protective factor for young people’s mental health [15]. Despite this, evidence suggests that those experiencing higher levels of suicidal or self-harming thoughts and behaviors are less likely to seek help for their mental health difficulties [16].

Growing use of computer-mediated technologies and web-based resources have changed the nature of help-seeking, making it possible for users to engage in help-seeking behaviors without an interpersonal component [10]. While offline resources remain an important source of help to young people, the accessibility of the Internet has created an opportunity for more sources of help and information to become available [17,18]. A study by Dooley & Fitzgerald [15] indicated that 77% of young people were likely to use the Internet to find information or support for a mental health concern. The increased role of alternative sources of help, such as YouTube, bloggers or Influencers, self-help websites and discussion forums, have to be considered [12]. There is a need to investigate how young people use the Internet as part of their help-seeking strategies and how they can be supported in these strategies.

Although many web-based information resources and interventions are available, they are of varying quality [19]. A study by Feng et al [20] found that while there are many online resources available, this does not necessarily result in user engagement or show that their use is helpful to the help-seeking process. The amount of evidence regarding the usefulness of online resources in facilitating the help-seeking process is a notable gap in the literature [21].

As with offline help-seeking, each young person has their own preferences for both online sources and preferred pathways in order to cope with their mental health difficulties and concerns [22]. Thus, the need to identify these sources and why they are attractive to young people is important. The aim of this study was to investigate and better understand the online help-seeking behaviors of young people. This was achieved through an online survey addressing several key issues, including current areas of concern, intentions to seek help, preferred online resources, credibility of online resources, and finally the current wellbeing of this sample.

## Methods

### Overview

Ethics approval for this research was provided by the University College Dublin Office of Research Ethics (LS-17-116-Pretorious-Coyle). All data was collected through an anonymous online survey.

### Survey Development

This survey was undertaken with the support of a youth mental health charity, ReachOut Ireland, who are the sister organization of ReachOut Australia. ReachOut is a mental health service that offers online mental health resources specifically for young people, but they also run a youth participation program that ensures young people’s involvement through all their work. Prior to the survey going live, it was piloted with five young people from the ReachOut Ireland youth panel to hear their thoughts on the survey and its acceptability. This survey was developed iteratively and informed by research in the area [13,17,23,24], and along with input and previous research from ReachOut Ireland [25] and the commentary from the youth panel, it was made as accessible and nonthreatening to as many young people as possible. In adhering to this input from the youth panel, the final survey did not refer specifically to symptoms such as feeling anxious or having a low mood and instead asked young people about the personal concerns that were causing them the most stress or worry. The term personal or emotional concern was selected, as the authors wanted to use nonmedicalized language throughout the survey. The concerns addressed in the survey also represent the most frequently expressed concerns on the ReachOut Ireland website.

### Survey Procedure

This study made use of a survey link to direct participants to the survey. This link was made available through various online sources, such as youth mental health-related websites (ReachOut Ireland, SpunOut, and BodyWhys), and through targeted advertisements posted on Facebook and Twitter. The adverts consisted of a short title, an image, and the survey link. The Facebook and Twitter advertisements were specifically targeted to appear on the feeds of Irish users between the ages of 18 and 25. The survey was hosted on LimeSurvey on a local server. The first component of the survey consisted of the information page, which included information regarding the purposes of the study, how the data would be used, anonymity, confidentiality and data protection. Participants were then asked to provide consent and confirm that they were both between the ages of 18-25 years old and living in Ireland, if they wished to continue with the survey. Information on mental health support was provided on the landing page of the survey as well as on the survey termination page. The survey consisted of 22 questions, over 6 screens, and took between 15 and 20 minutes to complete (see Multimedia Appendix 1 for the survey questions). Multiple responses from the same user were prevented by using cookies, and no incentive was offered. Participants were permitted to skip any question they were unwilling to answer during the survey. In total, 2352 people began the survey, but a total of only 1308 participants successfully completed the entire questionnaire. Data from uncompleted surveys was not used as withdrawal from the survey indicated withdrawal of consent.

### Survey Measures

The survey consisted of both quantitative and qualitative questions to assess: (1) demographics; (2) young people’s technology use; (3) propensity to seek help from different sources as measured by the General Help-Seeking Questionnaire (GHSQ) [23]; (4) current personal and emotional concerns; (5) preferred online resources; (6) credibility of online resources; (7) facilitators and barriers to online help-seeking; and (8) wellbeing of participants measured by the Short Warwick–Edinburgh Mental Well-being Scale (SWEMWBS) [24].This paper will discuss findings from (2), (4), (5), (6), and (7) in detail, and findings from (3) and (8) are included under the description of the survey participants.

### Data Analysis

The survey data were analyzed using IBM SPSS Statistics for Mac, Version 24, (IBM Corporation, Armonk, NY) primarily using descriptive statistics. Only completed surveys were analyzed. Open response questions were analyzed using thematic analysis [26].

## Results

### Survey Participants

A total of 1308 participants were examined in this study, of which 78.52% (1027/1308) were female, 18.50% (242/1308) were male, 1.68% (22/1308) were non-binary and 0.84% (11/1308) identified as transgender. The mean age of the population was 20.68 (SD 2.22), with a minimum and maximum age of 18 and 25, respectively*.* The survey had good national coverage, with respondents from all Irish counties. Of the whole sample, 67.13% (878/1308) reported that they were currently living in a city or town, 59.17% (774/1308) reported their current level of education to be undergraduate, and most of the sample accessed the survey link through Twitter (68.88%; 901/1308) and Facebook (24.08%; 315/1308). The results from the GHSQ were like findings from previous studies, with a very high propensity for respondents to not seek help at all (45.2%; 591/1308). Informal sources of help were preferred over formal sources. Only 18.5% (242/1308) of the sample indicated that they would be likely or extremely likely to seek help from a mental health professional, whereas 53.3% (697/1308) of the sample were likely or extremely likely to seek help from an intimate partner (see Multimedia Appendix 2). The SWEMWBS has good internal consistency, with a reported Cronbach alpha=0.84. In the current study, the Cronbach alpha=0.835. This sample mean (19.0362; SD 3.522) is one standard deviation lower than the normative group mean (23.6093; SD 3.90264). Scores on the SWEMWBS can range from 7 to 35, and higher scores on the SWEMWBS indicate higher positive mental well-being.

### Young People’s Technology Use

Most respondents owned a mobile phone (99.62%; 1303/1308) and a laptop or computer (92.35%; 1208/1308), with fewer owning a tablet (38.91%; 509/1308) or gaming console (34.10%; 446/1308). Mobile phones were the preferred device for using the Internet in order to look for help online (80.66%; 1055/1308), with only 32.65% (427/1308) of the sample indicating that they would use their laptop or computer. A negligible proportion of the sample used a tablet or games console to access the Internet or to look for help (see Multimedia Appendix 1).

### Areas of Personal and Emotional Concern and Online Help-Seeking

The closed response questions indicated that school or college was a source of personal concern for most of the sample, with 87.08% (1139/1308) indicating that it had recently caused them stress. This was followed by concern caused by body image (73.01%; 955/1308) and exams (72.02%; 942/1308). These results are like ReachOut’s previous findings, which also found exams, school and body image to be major stressors for young people [25]. Table 1 lists the other triggers of stress responded to by respondents.

In the open response section of these questions, 100 respondents provided additional data. These concerns were grouped into the following themes: mental health, work, finances, harm from others, housing, sports, identity, interpersonal difficulties, parenting, physical health, transitional challenges and societal concerns. Table 2 outlines each theme with a quote taken from the survey as an example for each.

This question was followed by a question asking whether young people had gone online to look for help for these concerns. For this question, 85.32% (1116/1308) of the sample had gone online to look for help with their stress caused by school or college, 70.41% (921/1308) had gone online to look for help with concerns over body image, and 71.25% (932/1308) had looked for help with exams. In addition, most of the respondents, 85.78% (1122/1308), had also gone online to look for help with deciding on a career.

Respondents were asked if they had ever gone online to look for help for a family member or friend. A total of 68.43% (895/1308) indicated that they had gone online to look for help or information for a friend, while 55.58% (727/1308) indicated they had searched for help for a family member.

**Table 1:** Areas of personal or emotional concern (N=1308). All values are listed as n (%).

Stressor	Caused significant stress (yes)	Looked online for help (yes)
School or College	1139 (87.08)	1116 (85.32)
Body Image	955 (73.01)	921 (70.41)
Exams	942 (72.02)	932 (71.25)
Family	678 (51.83)
Money	888 (67.89)	727 (55.58)
Deciding on a career	859 (65.67)	1122 (85.78)
Relationships	830 (63.46)	838 (64.07)
Friends	808 (61.77)	763 (58.33)
Social Media	524 (40.06)	485 (37.08)
Illness of family member or friend	513 (39.22)	797 (60.93)
Local or World News	400 (30.58)	815 (62.31)
Personal Illness	370 (28.29)	858 (65.60)
Bullying	367 (28.06)	312 (23.85)
Sexuality	312 (23.85)	502 (38.40)

**Table 2:** Personal or emotional concerns qualitative responses.

Theme	Illustrative Quote
Mental Health	*Just mental health, specially general anxiety.* [P1011]
Finances	*Unemployment after 4 years in college.* [P1926]
Work	*Participating in a work environment, ie: an office or the service industry*. [P622]
Housing	*Living in rented accommodation- cost, relations with house mates.* [P1242]
Sports	*Competitive sport*. [P1812]
Identity	*Developing a sense of identity, trying to be the best*. [P1097]
Interpersonal Difficulties	*an ability to understand people, the fear of not accepted as a member of a group or have actual friends, fear of trust due to let downs*. [P938]
Parenting	*Being a parent.* [P203]
Physical Health	*Physical health (no diagnosed illness).* [P2109]
Transitional Challenges	*The process of finishing college and transitioning from a world where others organised so much of my life to having to find a job and be the only one with the responsibility to progress my life.* [P732]
Societal Concerns	*Guilt about seeing world atrocities such as the homelessness crisis and racism/sectarianism and not being able to do much about it.* [P376]

### Young People’s Preferred Online Resources

Respondents were asked which online sources they use to gain more information for personal or emotional concerns, and 82.57% (1080/1308) indicated that they would make use of an Internet search, 57.03% (746/1308) indicated that they would use a health website, and 32.26% (422/1308) indicated they would make use of a forum or discussion board. Fewer (12.16%; 159/1308) would use a mental health app or go to a social media blogger or influencer (8.18%; 107/1308). An Internet search was widely used across all gender groups, while the use of a blogger or influencer was low across all groups.

In the open response section of this question, other preferred sources of information identified by the respondents could be grouped in the following ways: formal offline source, informal offline source, formal online source and informal online source. Examples of formal online resources included SpunOut.ie and ReachOut Ireland, while informal online sources included Reddit, YouTube and Tumblr.

In the subsequent question, respondents were asked how satisfied they were with their experiences of these sources if they had used them. The previous question indicated that the most preferred online resource by young people was the Internet search, and this question indicated that 36.94% (399/1080) of respondents were satisfied or very satisfied with an Internet search. The second most used online resource was a health website, and in this question 49.33% of respondents (368/746) indicated that they were satisfied or very satisfied with this resource (Table 3).

**Table 3:** Levels of satisfaction with online resources. All values listed as n (%).

Resource	Not sure	Very dissatisfied	Dissatisfied	Neutral	Satisfied	Very satisfied
Health website (N=746)	53 (7.1)	4 (0.54)	74 (9.92)	247 (33.11)	344 (46.11)	24 (3.22)
Mental health app (N=159)	19 (11.95)	4 (2.52)	22 (13.84)	55 (34.59)	52 (32.7)	7 (4.4)
Internet search (N=1080)	104 (9.63)	12 (1.11)	125 (11.57)	440 (40.74)	370 (34.26)	29 (2.69)
Influencer or blogger (N=107)	9 (8.41)	3 (2.8)	2 (1.87)	31 (28.97)	50 (46.72)	12 (11.21)
Forums or discussion board (N=422)	21 (4.97)	3 (0.71)	34 (8.06)	186 (44.08)	155 (36.73)	23 (5.45)
Websites already used (N=136)	10 (7.35)	2 (1.47)	17 (12.5)	62 (45.59)	38 (27.94)	7 (5.15)

### Credibility of Online Sources

Respondents were also asked how they would rate the trustworthiness of the above online resources. Health websites were found to be the most trustworthy, with 39.45% (516/1308) of respondents indicating that they found them trustworthy or very trustworthy. Respondents did not rate an Internet search as very trustworthy, with 47.09% (616/1308) saying that it was not trustworthy or only slightly trustworthy. Overall, none of the online sources listed were rated as trustworthy or very trustworthy by a majority of the respondents (Table 4). Following on from this question, respondents were asked which elements of an online resource would make it more credible. The vast majority (82.95%; 1085/1308) indicated that a health service logo was an important indicator of credibility, but an endorsement by schools and colleges (54.97%; 719/1308) or the presence of another government logo (57.57%; 753/1308) also played important roles (Table 5). Many respondents, specifically 80.43% (1052/1308), indicated that references to scientific data and authors were a key indicator of credibility in an online resource.

These indicators were followed by an open response question that asked, “Is there anything not listed above that makes an online resource trustworthy/reliable?”. In this section, respondents indicated that online security was important, citing elements such as the green padlock in the Internet browser as well as the lack of ads on webpages. Other themes identified in this open response section included: written or informed by a reputable person or organization, links to local support services, grounded in research, design and layout, quality of content, the ability to rank or comment on content, and the ability to contact someone directly through the source. Participants also mentioned cross-checking sources with other sources to ensure reliability and credibility of that source, as stated by one participant:

If its consistent with other online resources. If 3 or 4 sites say the same thing, they I begin to trust it.

**Table 4:** Trustworthiness of online resources (N=1308). All values listed as n (%).

Resource	Not trustworthy	Slightly trustworthy	It’s OK	Trustworthy	Very trustworthy	Don’t know
Health website	21 (1.61)	247 (18.88)	441 (33.72)	423 (32.34)	93 (7.11)	83 (6.35)
Mental health app	19 (1.45)	135 (10.32)	302 (23.09)	295 (22.55)	43 (3.29)	514 (39.30)
Internet search	163 (12.46)	453 (34.71)	482 (36.85)	123 (9.40)	3 (0.23)	84 (6.42)
Influencer or blogger	466 (35.63)	311 (23.78)	137 (10.47)	71 (5.43)	9 (0.69)	314 (24.01)
Forums or discussion board	222 (16.97)	395 (30.20)	285 (21.79)	126 (9.63)	13 (0.99)	267 (20.41)
Website already used	422 (32.26)	295 (22.55)	172 (13.15)	69 (5.28)	2 (0.15%)	348 (26.22)

**Table 5:** Elements that indicate credibility (N=1308). All values are listed as n (%).

Element	Disagree	Not sure	Agree
Links to social media	698 (53.36)	480 (36.70)	130 (9.94)
Government logo	254 (19.42)	301 (23.01)	753 (57.57)
Health service logo	82 (6.27)	141 (10.78)	1085 (82.95)
Good design and layout	434 (33.18)	334 (25.6)	540 (41.3)
Top of Google search results	460 (35.2)	331 (25.54)	517 (39.53)
College or school endorsement	190 (14.53)	399 (30.50)	719 (54.97)
References to scientific data and authors	64 (4.89)	192 (14.68)	1052 (80.43)
A quiz or assessment	618 (47.25)	474 (36.24)	216 (16.51)
Contains personal stories or experiences	177 (13.53)	433 (33.10)	698 (53.36)

Another participant suggested that sources could make this cross-checking process easier by providing hyperlinks to related work.

### Facilitators and Barriers to Seeking Help Online

Respondents were asked which factors would encourage them to seek help online if they were facing a personal or emotional concern (Table 6). Young people affirmed that the anonymity and confidentiality offered by the Internet was an important motivating factor when deciding to search for help online, with 80% (1046/1308) of the sample indicating that it influenced their decision a lot or quite a lot. Similarly, the low monetary cost of using the Internet was also an important motivator in selecting the Internet, with 84.41% (1104/1308) indicating it encouraged them to seek help online a lot or quite a lot. Some of the important barriers highlighted by young people included being unsure if information was reliable, as well as wanting to solve problems on their own (Table 7). Even though the Internet offers more anonymity than offline pathways, young people are still concerned about others finding out that they are experiencing a difficulty.

In the open response section, respondents were asked “Is there anything not listed above that would encourage you to seek help online for a personal or emotional concern?”. A total of 124 respondents provided answers. These answers were grouped together in themes, including anonymity, reduced stigma, validation of experiences, current situation in the health service and ease of access (Table 8). Respondents also highlighted barriers to online help-seeking in this space, such as the cost of some online services, lack of surety of credibility of some online resources, not being able to find personalized information, and a lack of mental health literacy which impacted their ability to find the right online resource (Table 8).

**Table 6:** Facilitators to online help-seeking (N=1308).

Facilitator	Mean (SD)	Not at all, n (%)	A little, n (%)	A lot, n (%)	Quite a lot, n (%)
It’s free	3.31 (0.81)	43 (3.29)	160 (12.23)	452 (34.56)	652 (49.85)
Anonymous and confidential	3.26 (0.89)	67 (5.12)	195 (14.91)	378 (28.90)	668 (51.07)
Can take it at own pace	3.16 (0.82)	47 (3.59)	205 (15.67)	547 (41.82)	509 (38.91)
Abundance of information	3.12 (0.80)	38 (2.91)	231 (17.66)	574 (43.88)	465 (35.55)
Others like me	3.10 (0.91)	74 (5.66)	257 (19.65)	437 (33.41)	540 (41.28)
Access any time of day	3.01 (0.88)	63 (4.82)	305 (23.32)	494 (37.77)	446 (34.10)
Unsure if I’m unwell enough	2.75 (1.09)	230 (17.58)	291 (22.25)	367 (28.06)	420 (32.11)
Too unwell to reach local support services	2.20 (1.04)	409 (31.27)	421 (32.19)	281 (21.48)	197 (15.06)
There are no other options available	2.13 (1.04)	447 (34.17)	436 (33.33)	238 (18.20)	187 (14.30)

**Table 7:** Barriers to online help-seeking (N=1308).

Barrier	Mean (SD)	Not at all, n (%)	A little, n (%)	A lot, n (%)	Quite a lot, n (%)
Unsure if information is reliable	2.70 (0.91)	111 (8.49)	465 (35.55)	440 (33.64)	292 (22.32)
Solve problems on my own	2.58 (1.09)	277 (21.18)	342 (26.15)	344 (26.30)	345 (26.38)
Concerns others might find out	2.36 (1.17)	417 (31.88)	329 (25.15)	230 (17.58)	332 (25.38)
Thinking I don’t have a problem	2.34 (1.04)	324 (24.77)	446 (34.10)	307 (23.47)	231 (17.66)
Unsure what to search for	2.13 (0.92)	348 (26.61)	570 (43.58)	261 (19.95)	129 (9.86)
Not sure of my privacy and anonymity	2.10 (1.05)	472 (36.09)	416 (31.80)	235 (17.97)	185 (14.14)
Prefer alternative forms of help	1.88 (0.94)	555 (42.43)	453 (34.63)	197 (15.06)	103 (7.87)
Having no one help navigate options	1.84 (0.97)	630) 48.17%	365 (27.91)	205 (15.67)	108 (8.26)
Being too unwell to look for help	1.73 (0.94)	703 (53.75)	351 (26.83)	155 (11.85)	99 (7.59)
Having previous bad experiences	1.61 (0.88)	786 (60.09)	320 (24.46)	125 (9.56)	77 (5.89)

**Table 8:** Qualitative responses indicating facilitators and barriers to online help-seeking.

Theme		Quote
**Facilitators**		
	Affordability	*Not having enough money to afford counselling in person*. [P889]
	Anonymity	*Some issues can feel embarrassing to talk about. The anonymity online cancels this out.* [P617]
	Ease of Access	*Its mostly just the speed of it that helps me out. In my case I can find so much info. on social anxiety with just one click rather than driving 30 minutes from my college to speak to the college counsellor.* [P1412]
	Validation of Experience	*Thinking that you are making up the illness in your head and that it isn't real and you are putting it on for attention*. [P1018]
	Reduced Stigma	*I often go online because I know there is something wrong, but I don't want to tell anyone in my real life for fear that they will judge me or they won't care and ill just be bothering them. Online help can help me deal with my problem alone so I will not have to tell anyone.* [P470]
	Privacy	*It provides a level of privacy and I feel like I can control my own feelings.* [P392]
	Response to negative life events	*A huge trauma maybe*. [P33]
**Barriers**		
	Affordability	*Hard to find a free option for when times are really bad.* [P1401]
	Lack of personalization	*It’s not personal: all the information out there already exists and is not tailored for me*. [P132]
	Lack of mental health literacy	*Being unsure what to search/look for online instead of searching for hours for a website that I am comfortable with*. [P242]
	Unsure of credibility	*Something to reassure me that the content I am viewing is reliable and trustworthy and having a person to discuss issues with*. [P863]

## Discussion

### Primary Findings

The results of this survey clearly indicate that the Internet plays a major role in the help-seeking process for young people. The survey has highlighted that young people are already going online to look for help for issues that are causing them distress, and they are engaging with different online sources for their help-seeking needs. Given the proportion of young people who encounter mental health difficulties and turn to the Internet to meet some of their mental health needs, it is important that researchers and service providers have an accurate and holistic understanding of what these needs encompass.

Help-seeking is a complicated process, and young people use different online mental health resources based on their needs. Rickwood’s model [27] of help-seeking refers to 4 stages of help-seeking: (1) becoming aware of and appraising the problem; (2) expressing the need for support; (3) knowledge of available and accessible sources of help; and (4) being willing to disclose personal information. This model acknowledges that there are several barriers that may impede help-seeking at any stage. It can be hypothesized that different online mental health resources are used at different stages of this process. Most of the sample, 82.6%, indicated that they would make use of an Internet search to locate information when experiencing a personal or emotional difficulty. The Internet search could be conceptualized as playing a role in both the expression and availability stages of the process. However, only 37% of the sample indicated that they were satisfied with this mode of finding help. This could indicate that the Internet search is being used due to its easily accessible nature and the anonymity it offers, but this appears insufficient to meet the mental health needs of the present sample. For these reasons, it is possible that an Internet search could act as both a facilitator and a barrier to further help-seeking.

Like findings in a study by Reavley, Cvetkovski & Jorm [22], health websites and discussion boards or forums seem to play an important role in meeting young people’s mental health needs, which may be due to varied reasons. A health website is likely to provide more accurate information substantiated by research and written by subject experts, while forums allow users to engage with peers who are like them and have lived their same experiences. Comparably, a study by Lal, Nguyen & Theriault [28] indicated that young people value resources that allow them to access the personal stories of peers with lived experiences, which gives them the opportunity to process the information at their own pace. The current study found that other popular online resources include formal youth mental health websites, such as ReachOut Ireland, and informal sites such as YouTube. It is worth noting that these sources are likely to change with time and new or other platforms grow in popularity.

Young people are often described as digital natives [29]. This includes the assumption that young people can effectively identify and locate credible resources in the online space [30]. A study by Montagni et al [31] found that half of their sample trusted what they found on the Internet, but their sample identified one of the disadvantages of using the Internet was its unreliability. This survey also indicates that assigning online credibility can be confusing, but young people have developed different strategies to determine the reliability of an online resource. Some of these strategies include checking multiple sources and cross-checking information. The results from this survey have shown endorsements from reputable and known government bodies and educational institutions can play an important role in helping young people to identify credible and reliable online resources. It is evident, though, that the sources young people were surveyed on, apart from health websites, are not deemed to be very credible or reliable. This disparity between a plethora of online sources being available and their perceived lack of credibility could have a jarring effect on the help-seeking process of the young person, so this needs to be investigated.

There are a multitude of facilitators and barriers associated with online help-seeking. Many studies have found that the ease of access of the Internet plays an important role in helping young people, a finding that this study supports [24,32-34]. Particularly, a study by Birnbaum et al [35], highlighted that the Internet plays an important role in early intervention and in young people’s further help-seeking. Young people are drawn to the Internet because of the wealth of free resources available, but further help-seeking, such as talking to a professional, may be too costly for this demographic. A systematic review by Kauer, Mangan & Sanci [21] confirmed that online help-seeking is attractive to many young people because of its confidentiality and anonymity. This survey found that concerns about anonymity and privacy remain, and although it seems that the anonymity offered by the Internet does go a long way in circumventing the stigma associated with mental health help-seeking, young people are still concerned about others finding out. It may be for this reason that many of the sample indicated that they would use their mobile phone to search for help online.

### Limitations

The survey findings were based on self-reported data from the respondents, so the results might not be generalizable. Given that recruitment of participants happened through online platforms, this sample is limited to young people who access Facebook, Twitter and other charity websites. Thus, this survey may not have captured the views of help-seekers who access alternative resources on the Internet. Future studies should include alternative recruitment strategies targeting those who are less likely to seek help, particularly men, and help-seekers who may not access mainstream social media platforms or charity websites. In addition, a large majority of the participants were female and undergraduate students, which also limits the generalizability of the results. This survey focused on emotional concerns that cause participants significant distress for which they might go online to look for help but did not ask about searches for symptoms such as feeling depressed or feeling anxious. Thus, it cannot comment on the types of mental health symptoms participants might seek help for online. This should be taken into consideration in future work. Finally, the list of online resources offered was not extensive, so future studies should further investigate both the preferences between online and offline sources and whether there are potential differences between preferences for informal and formal online resources.

### Conclusion

The findings of this study indicate that young people are engaging in help-seeking behavior online to look for help for personal and emotional concerns that are causing them distress. Levels of satisfaction regarding different online resources are varied, so web-based mental health resources need to ensure that they meet the needs of online help-seekers in providing support. Young people have established strategies to assign credibility online, however, the availability of credible, online resources needs to be addressed. Steps should also be taken to help governmental organizations and educational bodies identify and support trustworthy and reliable online resources. Finally, as with traditional offline help-seeking, several barriers exist to deter help-seeking; however, the Internet circumvents some of these through its offering of privacy and confidentiality.

## Appendix

Multimedia Appendix 1 Survey Questions.

Multimedia Appendix 2 Additional Tables.

## Abbreviations

GHSQ: General Help-Seeking Questionnaire
SWEMWBS: Short Warwick–Edinburgh Mental Well-being Scale
