# Supplementary material for: Young People Seeking Help Online for Mental Health: Cross-Sectional Survey Study
Source: JMIR Ment Health. 2019 Aug 26;6(8):e13524. doi: 10.2196/13524 (PMC6732968; doi:10.2196/13524)
Supplement: Multimedia Appendix 1 [file mental_v6i8e13524_app1.pdf]

Information Sheet

Researcher: Ms. Claudette Pretorius

Supervisor: Dr David Coyle

School of Computer Science

University College Dublin

Funder: European Union's Horizon 2020 research and innovation programme under the Marie Sklodowska-Curie Action grant number 722561

Email: claudette.pretorius@ucdconnect.ie

Project Title: A Survey of Online Help-Seeking in Young Adults

This research is being conducted by Claudette Pretorius, a doctoral candidate in School of Computer Science at the University College of Dublin under the supervision of Dr. David Coyle. This research is part of the TEAM (Technology Enabled Youth Mental Health) consortium. Our focus is on the design and development of new technology-enabled mental health services.

We would like to invite you to take part in an online survey that looks at how young people look for information and help online for personal and emotional concerns. Before you decide whether to take part it is important that you understand why the research is being done and what it will involve. Please take time to read this information sheet carefully.

What is this research about?

This research is interested in learning how young people look for help for personal and emotional concerns online and which factors influence this process. We are particularly interested in your views of e-mental health and use of the internet and technology for mental health information and support.

Why are we doing this research?

We understand that it is sometimes difficult to find the right information online. By gathering information on young people's current online information and help-seeking behaviours, we hope by the end of the research project to develop an online tool that

Section A: Consent Form

A1. **I confirm that I am 18 years or older**

Yes ☐
No ☐

A2. **I have read and understood the information sheet on the previous page**

Yes ☐
No ☐

A3. **I understand that participation is voluntary and I can withdraw whilst taking the survey. I accept that once the survey is completed, I cannot withdraw due to the anonymous nature of the survey.**

Yes ☐
No ☐

A4. **I agree my anonymised research data will be stored securely by the researcher up 5 years after the completion of the project at which time the information will be destroyed securely.**

Yes ☐
No ☐

A5. **I consent to my anonymised data may be quoted in dissemination activities including but not limited to PhD thesis, scientific publication, conference proceedings and presentations**

Yes ☐
No ☐

A6. **I agree that my anonymised data can be stored in an Open Access repository**

Yes ☐
No ☐

A7. **I understand that all information I provide for this study will be treated confidentially where normal limits of confidentiality apply.**

Yes ☐
No ☐

A8. **I am aware that I can contact the researcher at any point to get further information or clarification about this research study.**

Yes ☐
No ☐

A9. **I am aware that when the research study is complete, findings or results from this research study will be available from the researcher.**

- [ ] Yes
- [ ] No

A10. **If you do NOT wish to take part in the study you can click away from the survey now.**

If you do wish to take part tic the box "I consent to take part in the study" and then click on.

- [ ] I consent to take part in the study

Section B: Demographics

This section of the survey would like to determine some demographic details from you.

B1. **Please indicate your gender**

- [ ] Male
- [ ] Female
- [ ] Transgender
- [ ] Non-binary
- [ ] Prefer not to say

B2. **What is your current age?**

- [ ] 18
- [ ] 19
- [ ] 20
- [ ] 21
- [ ] 22
- [ ] 23
- [ ] 24
- [ ] 25

B3. **In which county do you live?**

- Carlow ☐
- Cavan ☐
- Clare ☐
- Cork ☐
- Donegal ☐
- Dublin ☐
- Galway ☐
- Kerry ☐
- Kildare ☐
- Kilkenny ☐
- Laois ☐
- Leitrim ☐
- Limerick ☐
- Longford ☐
- Louth ☐
- Mayo ☐
- Meath ☐
- Monaghan ☐
- Offaly ☐
- Roscommon ☐
- Sligo ☐
- Tipperary ☐
- Waterford ☐
- Westmeath ☐
- Wexford ☐
- Wicklow ☐

B4. **Do you live in a...**

- City / Town ☐
- Rural area ☐

B5. What is your current level of education?

- [ ] 5th Year
- [ ] 6th Year
- [ ] Undergraduate
- [ ] Postgraduate
- [ ] PhD
- [ ] Apprenticeship
- [ ] Other

Other

Section C: Areas of Stress

C1. Please indicate if any of the following have caused you a lot of stress

- [ ] School / College
- [ ] Family
- [ ] Friends
- [ ] Social Media
- [ ] Money
- [ ] Body Image
- [ ] Exams
- [ ] Relationships
- [ ] Sexuality
- [ ] Bullying
- [ ] Local or world news
- [ ] Deciding on a career
- [ ] Personal Illness
- [ ] Illness of a family member or friend

C2. **Has anything that is not on the list above caused you stress or difficulty?**

C3. **Can you rank your top 3 causes of stress?**

School / College	
Family	
Friends	
Social Media	
Money	
Body Image	
Exams	
Relationships	
Sexuality	
Bullying	
Local or world news	
Deciding on a career	
Personal Illness	
Illness of a family member or friend	

Section D: Information and help-seeking

D1. On an average day, if you were having a personal or emotional problem, how likely are you to seek help from the following people? Please mark the appropriate response

	Extremely Unlikely	Unlikely	Neutral	Likely	Extremely likely
Friend (not related to you)	☐	☐	☐	☐	☐
Parent	☐	☐	☐	☐	☐
Other relative/family member	☐	☐	☐	☐	☐
Intimate partner (e.g. girlfriend, boyfriend, partner, husband, wife)	☐	☐	☐	☐	☐
GP / family doctor	☐	☐	☐	☐	☐
Mental health professional (e.g. psychologist, social worker, counsellor)	☐	☐	☐	☐	☐
Teacher / school counsellor	☐	☐	☐	☐	☐
Minister or religious leader (e.g. priest, rabbi, chaplain)	☐	☐	☐	☐	☐
Phone helpline (e.g. Samaritans)	☐	☐	☐	☐	☐
Online counselling service (e.g. Niteline)	☐	☐	☐	☐	☐
A person I know has had their own experiences of emotional/mental health difficulties	☐	☐	☐	☐	☐
Blogger / influencer	☐	☐	☐	☐	☐
I would not seek help from anyone	☐	☐	☐	☐	☐

D2. Have you ever gone online to learn more about a personal or emotional problem you or someone close to you was experiencing?

	Yes	Uncertain	No
Family member	☐	☐	☐
Friend	☐	☐	☐

D3. **Please indicate if you've ever gone online to look for help or information for any of the following?**

	Yes	Uncertain	No
School / College	☐	☐	☐
Family	☐	☐	☐
Friends	☐	☐	☐
Social Media	☐	☐	☐
Money	☐	☐	☐
Body Image	☐	☐	☐
Exams	☐	☐	☐
Relationships	☐	☐	☐
Sexuality	☐	☐	☐
Bullying	☐	☐	☐
Local or world news	☐	☐	☐
Deciding on a career	☐	☐	☐
Personal Illness	☐	☐	☐
Illness of a family member or friend	☐	☐	☐

D4. **Please tick the appropriate responseDo you own any of the following devices?**

- Smartphone ☐
- Tablet ☐
- Laptop / Computer ☐
- Games Console ☐

D5. **Please tick the appropriate responseWhich device do you use the most to access the internet?**

- Smartphone ☐
- Tablet ☐
- Laptop / Computer ☐
- Games Console ☐

D6. **Please tick the appropriate responseWhich device would you use to access the internet to look for information/help for personal and emotional concerns?**

- [ ] Smartphone
- [ ] Tablet
- [ ] Laptop / Computer
- [ ] Games Console

D7. **If you wanted more information about a personal or emotional problem concern, where would you find more information?**

- [ ] Health website
- [ ] Mental Health App
- [ ] Internet search
- [ ] Social media￼Blogger/ Influencer
- [ ] Forums / discussion boards
- [ ] Websites that you already use for other kinds of content e.g. Buzzfeed
- [ ] Other

Other

D8. **How satisfied were you with the information you found from this resource?**

	Not sure	Very dissatisfied	Dissatisfied	Neutral	Satisfied	Very satisfied
Health website	☐	☐	☐	☐	☐	☐
Mental Health App	☐	☐	☐	☐	☐	☐
Internet search	☐	☐	☐	☐	☐	☐
Social media￼Blogger/ Influencer	☐	☐	☐	☐	☐	☐
Forums / discussion boards	☐	☐	☐	☐	☐	☐
Websites that you already use for other kinds of content e.g. Buzzfeed	☐	☐	☐	☐	☐	☐

D9. Would you mind saying why?

We would like to know why you experienced these online sources the way you did. What do they get right and what do they do wrong.

Section E: Credibility of Online resources

E1. How trustworthy / reliable do you find the information online from the following resources:

	Don't know	Not trustworthy at all	Slightly trustworthy	It's OK	Trustworth y	Very trustworthy
Health website	☐	☐	☐	☐	☐	☐
Mental Health App	☐	☐	☐	☐	☐	☐
Internet search	☐	☐	☐	☐	☐	☐
Social media�Blogger/ Influencer	☐	☐	☐	☐	☐	☐
Forums / discussion boards	☐	☐	☐	☐	☐	☐
Websites that you already use for other kinds of content e.g. Buzzfeed	☐	☐	☐	☐	☐	☐

E2. Which of these features makes an online resource trustworthy/reliable?

	Disagree	Not sure	Agree
Links to social media	☐	☐	☐
Government logo	☐	☐	☐
HSE Logo	☐	☐	☐
Good design and layout	☐	☐	☐
At the top of a Google search results page	☐	☐	☐
College or school endorsement	☐	☐	☐
References to scientific data and authors	☐	☐	☐
A quiz or assessment	☐	☐	☐
Contains personal stories / experiences	☐	☐	☐

E3. Is there anything not listed above that makes an online resource trustworthy/reliable?

Section F: Motivations for online help-seeking

F1. How likely are the following factors to encourage you to seek help online or through an online resource for a personal or emotional concern?

	Not at all	A little	A lot	Quite a lot
I can access information and support online any time of day	☐	☐	☐	☐
Online help is mostly free	☐	☐	☐	☐
Going online is anonymous and confidential	☐	☐	☐	☐
An abundance of information is readily available	☐	☐	☐	☐
I can go through information and take action at my own pace	☐	☐	☐	☐
Knowing that there are others in the world who have gone through similar tough times	☐	☐	☐	☐
Being too unwell to reach out to my local support services	☐	☐	☐	☐
Being unsure if I am unwell enough to reach out to local support services	☐	☐	☐	☐
There are no other options available to me	☐	☐	☐	☐

F2. Have any of these issues ever stopped, delayed or discouraged you from seeking help online or through an online resource for a personal or emotional concern?

	Not at all	A little	A lot	Quite a lot
Being unsure what to search for online	☐	☐	☐	☐
Wanting to solve the problem on my own	☐	☐	☐	☐
Preferring to get alternative forms of help	☐	☐	☐	☐
Being unsure if the information I find is reliable	☐	☐	☐	☐
Being too unwell to look for help	☐	☐	☐	☐
Concern that others (family/friends) might find out	☐	☐	☐	☐
Having previous bad experiences with looking for help online	☐	☐	☐	☐
Thinking that I don't have a problem	☐	☐	☐	☐
Having no one who could help me navigate all the options online	☐	☐	☐	☐
Not being sure of my privacy and anonymity online	☐	☐	☐	☐

F3. Is there anything not listed above that would encourage you to seek help online for a personal or emotional concern?

F4. Is being able to connect to another person online important to you when seeking help online? If yes, why?

Section G: Wellbeing

G1. Below are some statements about feelings and thoughts.

Please tick the box that best describes your experience of each over the last 2 weeks.

"Short Warwick Edinburgh Mental Well-Being Scale (SWEMWBS) © NHS Health Scotland, University of Warwick and University of Edinburgh, 2008, all rights reserved."

	None of the time	Rarely	Some of the time	Often	All of the time
I've been feeling optimistic about the future	☐	☐	☐	☐	☐
I've been feeling useful	☐	☐	☐	☐	☐
I've been feeling relaxed	☐	☐	☐	☐	☐
I've been dealing with problems well	☐	☐	☐	☐	☐
I've been thinking clearly	☐	☐	☐	☐	☐
I've been feeling close to people	☐	☐	☐	☐	☐
I've been able to make up my own mind about things	☐	☐	☐	☐	☐

Thank you for taking the time to complete this survey!

If you would like to be kept informed about the survey and the broader study, please contact Ms. Claudette Pretorius on Claudette.Pretorius@ucdconnect.ie . You can also find more general information about the TEAM project at this web address: www.team-itn.eu

Support Services

The services listed below may be of benefit if you are looking for information or support.

Samaritans: Phone: 116 123 (Freephone, Republic of Ireland)

Email: jo@samaritans.org

24 hours a day, 365 days a year

If you are in crisis:

Contact your local doctor. To find yours, visit www.icgp.ie/go/find_a_gp Go to, or contact, the Accident and Emergency Department of your nearest general hospital Emergency services can be contacted at any time by dialling 999 or 112.

Mental health information and support services:

www.reachout.com - offers comprehensive information on all aspects of mental health and how to get help, and provides a safe space for engaging with a team of trained moderators and a panel of experts.

www.turn2me.org - provides a variety of support options, including online counselling, online support groups and live chat.

www.yourmentalhealth.ie – provides information on mental health, support services near you and the everyday #littlethings that can make a difference to your mental health and wellbeing.

www.bodywhys.ie – offers online support for eating disorder issues, including an online group counselling service.

www.drugs.ie – offers drug and alcohol information and support including a 'live helper' service that lets you chat to a staff member online.
