# Supplementary material for: Young People Seeking Help Online for Mental Health: Cross-Sectional Survey Study
Source: JMIR Ment Health. 2019 Aug 26;6(8):e13524. doi: 10.2196/13524 (PMC6732968; doi:10.2196/13524)
Supplement: Multimedia Appendix 2 [file mental_v6i8e13524_app2.pdf]

Table 1: SWEMWBS Results according to gender

	n	Mean	Std. Deviation	Std. Error
Male	242	19.4416	3.82944	.24617
Female	1027	18.9998	3.46647	.10817
Transgender	11	17.7791	3.03983	.91654
Non-binary	22	17.4218	2.24148	.47788
Prefer not to say	6	17.1517	1.70090	.69439
Total	1308	19.0362	3.52199	.09738

Table 2: GSHQ results

Help source (1=”extremely unlikely”; 5=”extremely likely”)	Mean (SD)	1 %(n)	2 %(n)	3 %(n)	4 %(n)	5 %(n)
Informal Offline Sources						
A friend	3.17(1.24)	11.1 (145)	23.6 (309)	15.1 (198)	37.4 (489)	12.8 (167)
A parent	2.58(1.32)	26.4 (345)	28.1 (368)	15.2 (199)	21.4 (280)	8.9 (116)
Another relative/family member	1.93(1.08)	44.9 (587)	31.7 (414)	10.6 (138)	11.0 (144)	1.9 (25)
Intimate partner (e.g. girlfriend, boyfriend, partner, husband, wife)	3.37(1.37)	15.2 (199)	11.1 (145)	20.4 (267)	28.1 (367)	25.2 (330)
Formal Offline Sources						
GP / family doctor	1.77(0.94)	50.9 (666)	28.7 (376)	13.6 (178)	6.3 (82)	0.5 (6)
Mental health professional (e.g. psychologist, social worker, counsellor)	2.08(1.19)	42.2 (555)	27.6 (361)	12.9 (169)	13.5 (176)	3.6 (47)
Teacher / school counsellor	1.65(0.92)	58.6 (766)	25.3 (331)	9.9 (130)	5.4 (71)	0.8 (10)

Minister or religious leader (e.g. priest, rabbi, chaplain)	1.15(0.45)	89.7 (1173)0.	7.0 (91)	2.2 (29)	1.1 (14)	0.1 (1)
Phone helpline (e.g. Samaritans)	1.51(0.81)	65.1 (852)	23.5 (307)	7.4 (97)	3.7 (48)	0.3 (4)
<u>Other</u>						
I would not seek help from anyone	3.15(1.25)	11.0 (144)	23.5 (308)	20.3 (265)	30.1 (394)	15.1 (197)

Table 3: Young people's technology use

	Own (yes)	Access Internet (yes)	To look for help (yes)
	n(%)	n(%)	n(%)
Mobile Phone	1303 (99.62%)	1232 (94.19%)	1055 (80.66%)
Laptop/Computer	1208 (92.35%)	246 (18.81%)	427 (32.65%)
Tablet	509 (38.91%)	43 (3.29%)	54 (4.13%)
Games Console	446 (34.10%)	12 (0.92%)	0 (0%)

Table 4: Preferred online resources

	Females (yes)	Males (yes)	Non-binary (yes)	Transgender (yes)	Prefer not to say (yes)	Total (yes)
N	1027	242	22	11	6	
Health website	602 (58.62%)	123 (50.83%)	13 (59.09%)	4 (36.36%)	4 (66.67%)	746 (57.03%)
Mental Health App	132 (12.85%)	24 (9.92%)	3 (13.64%)	0 (0%)	0 (0%)	159 (12.16%)
Internet Search	848 (82.57%)	195 (80.58%)	20 (90.91%)	11 (100%)	6 (100%)	1080 (82.57%)
Influencer /blogger	82 (7.98%)	21 (8.68%)	3 (13.64%)	1 (9.09%)	0 (0%)	107 (8.18%)
Forums / discussion board	323 (31.45%)	86 (35.54%)	10 (45.45%)	1 (9.09%)	2 (33.33%)	422 (32.26%)
Websites already used	118 (11.49%)	13 (5.37%)	4 (18.18%)	0 (0%)	1 (16.67%)	136 (10.40%)
